# Supplementary material for: Multiomics Analysis of a Micronutrient-Rich Dietary Pattern and the Aging Genotype 9p21 on the Plasma Proteome of Young Adults
Source: Nutrients. 2025 Apr 21;17(8):1398. doi: 10.3390/nu17081398 (PMC12030164; doi:10.3390/nu17081398)
Supplement: Supplementary file 1 [file nutrients-17-01398-s001.zip › nutrients-3576406-supplementary.pdf]

Supplementary Table S1. Average Protein Concentration by 9p21 genotype (rs2383206) and prudent dietary score (n=1,280).

	Prudent Dietary Score			p-value		
**Protein & Genotype**	**Low**	**Medium**	**High**	**Diet**	**Gene**	**Interaction**
**Adiponectin**				0.03	0.19	0.26
AA & GA	0.1 ± 0.0	0.1 ± 0.0	0.1 ± 0.0			
GG	0.1 ± 0.0	0.1 ± 0.0	0.1 ± 0.0			
**Afamin**				0.51	0.19	0.6
AA & GA	0.2 ± 0.1	0.2 ± 0.1	0.2 ± 0.1			
GG	0.3 ± 0.1	0.2 ± 0.1	0.2 ± 0.1			
**Albumin**				0.45	0.07	0.54
AA & GA	966.2 ± 143.4	974.9 ± 151.6	980 ± 167.6			
GG	978.2 ± 149.7	999.3 ± 163	1006.7 ± 154.9			
**α_1 Acid Glycoprotein***				0.11	0.02	0.15
AA & GA	1.7 ± 0.6	1.8 ± 0.7	1.8 ± 0.6			
GG	1.9 ± 0.7	1.9 ± 0.7	1.9 ± 0.9			
**α_1 Antichymotrypsin**				0.20	< 0.01	0.02
AA & GA	3.2 ± 0.7	3.4 ± 0.8	3.4 ± 0.8			
GG	3.6 ± 0.8	3.5 ± 0.9	3.6 ± 0.7			
**α_1 Antitrypsin**				0.25	0.01	0.48
AA & GA	9.9 ± 1.8	10.3 ± 2.2	10.2 ± 2.2			
GG	0.6 ± 2.4	10.6 ± 2.6	10.6 ± 2.4			
**α_{1B} Glycoprotein**				0.41	0.11	0.48
AA & GA	1.6 ± 0.5	1.6 ± 0.5	1.6 ± 0.5			
GG	1.6 ± 0.5	1.7 ± 0.5	1.6 ± 0.5			
**α_2 Antiplasmin**				0.7	0.03	0.40
AA & GA	1.9 ± 0.4	1.9 ± 0.4	1.9 ± 0.5			
GG	2.0 ± 0.4	2.0 ± 0.5	2.0 ± 0.4			
**α_2 HS-Glycoprotein**				0.83	0.03	0.32
AA & GA	8.3 ± 1.5	8.4 ± 2.0	8.3 ± 2.1			
GG	8.9 ± 1.7	8.6 ± 2.1	8.7 ± 1.6			
**α_2 Macroglobulin**				0.6	0.04	0.90
AA & GA	5.7 ± 1.5	5.8 ± 1.8	5.8 ± 1.7			
GG	5.9 ± 1.9	6.1 ± 1.8	6.2 ± 1.5			
**Angiotensinogen***				0.95	0.08	0.47
AA & GA	0.7 ± 0.2	0.7 ± 0.3	0.7 ± 0.2			
GG	0.7 ± 0.4	0.7 ± 0.2	0.7 ± 0.2			
**Antithrombin III**				0.28	0.05	0.38
AA & GA	3.5 ± 0.5	3.6 ± 0.6	3.6 ± 0.7			
GG	3.7 ± 0.6	3.7 ± 0.6	3.8 ± 0.6			
**Apolipoprotein A1**				0.10	0.68	0.81
AA & GA	41.4 ± 8.6	41.4 ± 8.8	42.8 ± 10.1			
GG	41.5 ± 9.5	41.7 ± 9.5	43.5 ± 9.8			
**Apolipoprotein A2**				0.68	0.13	0.53
AA & GA	23.5 ± 4.8	23.7 ± 5.1	24 ± 5.3			
GG	24.3 ± 4.1	24.2 ± 4.9	24.4 ± 5.4			
**Apolipoprotein A4**				0.04	0.03	0.24
AA & GA	1.4 ± 0.5	1.4 ± 0.5	1.5 ± 0.4			
GG	1.5 ± 0.5	1.5 ± 0.4	1.6 ± 0.5			
**Apolipoprotein B100**				0.48	0.22	0.31
AA & GA	0.8 ± 0.2	0.8 ± 0.2	0.8 ± 0.2			
GG	0.8 ± 0.2	0.8 ± 0.2	0.7 ± 0.2			
**Apolipoprotein CI**				0.57	0.05	0.44
AA & GA	3.1 ± 0.8	3.1 ± 0.9	3.1 ± 0.9			
GG	3.2 ± 0.8	3.3 ± 0.9	3.2 ± 0.8			
**Apolipoprotein CIII**				< 0.01	0.63	0.61
AA & GA	2.1 ± 0.7	2.2 ± 0.7	2.4 ± 0.8			
GG	2.1 ± 0.7	2.3 ± 0.9	2.3 ± 0.7			
**Apolipoprotein D**				0.66	0.99	0.48
AA & GA	0.3 ± 0.1	0.3 ± 0.1	0.3 ± 0.1			
GG	0.4 ± 0.1	0.3 ± 0.1	0.3 ± 0.1			

**Apolipoprotein E**				0.17	0.76	0.94
AA & GA	0.5 ± 0.2	0.5 ± 0.2	0.5 ± 0.2			
GG	0.5 ± 0.2	0.5 ± 0.2	0.5 ± 0.2			

	**Prudent Dietary Score**			**p-value**		
	**Low**	**Medium**	**High**	**Diet**	**Gene**	**Interaction**
**β_2 Glycoprotein 1**				0.02	0.03	0.12
AA & GA	2.7 ± 0.5	2.8 ± 0.6	2.8 ± 0.7			
GG	2.9 ± 0.6	2.8 ± 0.8	3.1 ± 0.7			
**Ceruloplasmin***				0.13	0.05	0.48
AA & GA	1.9 ± 0.5	2.0 ± 0.6	2.0 ± 0.6			
GG	2.1 ± 0.7	2.1 ± 0.8	2.0 ± 0.6			
**Clusterin**				0.15	0.19	0.92
AA & GA	1.5 ± 0.3	1.5 ± 0.3	1.5 ± 0.3			
GG	1.5 ± 0.3	1.5 ± 0.3	1.5 ± 0.3			
**Coagulation Factor XIIa**				< 0.001	0.01	0.99
AA & GA	0.2 ± 0.1	0.2 ± 0.1	0.3 ± 0.1			
GG	0.2 ± 0.1	0.2 ± 0.1	0.3 ± 0.1			
**Complement C1 inactivator**				0.43	< 0.01	0.81
AA & GA	4.9 ± 1	4.9 ± 1.1	4.8 ± 1.1			
GG	5 ± 1.1	5.2 ± 1.2	5 ± 1			
**Complement C3**				0.69	< 0.01	0.44
AA & GA	18.5 ± 3.8	19 ± 4.7	19 ± 4.6			
GG	20.6 ± 4.9	20 ± 6	19 ± 4.2			
**Complement C4 β chain**				0.45	< 0.01	0.05
AA & GA	1.4 ± 0.5	1.4 ± 0.5	1.4 ± 0.5			
GG	1.6 ± 0.6	1.4 ± 0.6	1.4 ± 0.6			
**Complement C4 γ chain**				0.59	< 0.01	0.02
AA & GA	1.5 ± 0.6	1.6 ± 0.6	1.5 ± 0.5			
GG	1.8 ± 0.7	1.7 ± 0.7	1.5 ± 0.7			
**Complement Factor B**				0.12	< 0.001	0.37
AA & GA	1.3 ± 0.3	1.4 ± 0.4	1.4 ± 0.4			
GG	1.5 ± 0.4	1.5 ± 0.5	1.5 ± 0.3			
**Complement Factor H**				0.29	0.01	0.05
AA & GA	0.6 ± 0.1	0.6 ± 0.2	0.6 ± 0.2			
GG	0.6 ± 0.2	0.6 ± 0.2	0.6 ± 0.2			
**Complement C9**				0.02	0.09	0.03
AA & GA	2.5 ± 0.9	2.7 ± 0.8	2.8 ± 0.9			
GG	2.8 ± 0.7	2.8 ± 1.0	2.8 ± 0.7			
**Fibrinogen α chain***				0.08	0.91	0.03
AA & GA	10.7 ± 4.5	12.2 ± 7.0	11.9 ± 5.8			
GG	11.6 ± 3.5	11.9 ± 4.4	11.8 ± 3.5			
**Fibrinogen β chain***				0.02	0.24	0.07
AA & GA	8.6 ± 3.2	9.7 ± 4.6	9.4 ± 3.9			
GG	9.5 ± 2.7	9.5 ± 3.4	9.5 ± 2.8			
**Fibrinogen γ chain***				< 0.01	0.28	0.24
AA & GA	8.5 ± 3.2	9.6 ± 4.9	9.4 ± 4.4			
GG	9.2 ± 3.1	9.7 ± 4.0	9.3 ± 3.0			
**Fibrinopeptide A***				0.02	0.18	0.02
AA & GA	6.4 ± 2.2	7.1 ± 3.5	7.0 ± 2.7			
GG	7.1 ± 2.2	7.1 ± 2.5	7.0 ± 1.9			
**Fibronectin***				0.07	0.77	0.74
AA & GA	0.5 ± 0.6	0.7 ± 1.4	0.6 ± 1.0			
GG	0.6 ± 0.8	0.6 ± 0.8	0.5 ± 0.5			
**Gelsolin**				0.49	0.17	0.99
AA & GA	1.2 ± 0.3	1.2 ± 0.3	1.2 ± 0.3			
GG	1.2 ± 0.3	1.3 ± 0.3	1.3 ± 0.3			
**Haptoglobin β chain**				0.33	< 0.01	0.13
AA & GA	10.3 ± 5.4	10.8 ± 5.8	10.2 ± 5.3			
GG	12.7 ± 5.1	11.3 ± 6.2	10.6 ± 4.3			
**Hemopexin**				0.04	< 0.01	< 0.01
AA & GA	9.5 ± 1.8	9.7 ± 2.0	10.0 ± 2.3			
GG	10.2 ± 2.0	9.9 ± 2.6	10.6 ± 2.1			

**Heparin cofactor II**				0.88	0.02	0.07
AA & GA	0.6 ± 0.2	0.7 ± 0.2	0.6 ± 0.2			
GG	0.7 ± 0.2	0.7 ± 0.2	0.7 ± 0.2			
**Histidine-rich glycoprotein**				0.08	0.14	0.20
AA & GA	1.3 ± 0.3	1.4 ± 0.4	1.4 ± 0.4			
GG	1.4 ± 0.4	1.4 ± 0.5	1.5 ± 0.4			

	**Prudent Dietary Score**			**p-value**		
	**Low**	**Medium**	**High**	**Diet**	**Gene**	**Interaction**
**Inter- α trypsin inhibitor HS**				0.79	0.02	0.47
AA & GA	0.6 ± 0.1	0.6 ± 0.1	0.6 ± 0.1			
GG	0.6 ± 0.1	0.6 ± 0.1	0.6 ± 0.1			
**Kininogen-1**				0.07	0.02	0.60
AA & GA	1.9 ± 0.3	2 ± 0.4	2 ± 0.4			
GG	2 ± 0.4	2.1 ± 0.5	2.1 ± 0.4			
**L-Selectin**				0.85	0.44	0.51
AA & GA	0.1 ± 0	0.1 ± 0	0.1 ± 0			
GG	0.1 ± 0	0.1 ± 0	0.1 ± 0			
**Plasma-retinol-BP**				< 0.001	0.27	0.39
AA & AG	0.8 ± 0.2	0.8 ± 0.2	0.9 ± 0.3			
GG	0.9 ± 0.2	0.9 ± 0.2	0.9 ± 0.2			
**Plasminogen**				0.8	0.01	0.13
AA & GA	1.1 ± 0.2	1.2 ± 0.2	1.2 ± 0.3			
GG	1.2 ± 0.2	1.3 ± 0.3	1.2 ± 0.2			
**Prothrombin**				0.66	0.08	0.2
AA & GA	0.6 ± 0.1	0.6 ± 0.1	0.6 ± 0.1			
GG	0.6 ± 0.1	0.6 ± 0.1	0.6 ± 0.1			
**Serum amyloid P-component**				0.18	0.001	0.001
AA & GA	0.4 ± 0.1	0.4 ± 0.2	0.4 ± 0.1			
GG	0.5 ± 0.1	0.5 ± 0.2	0.4 ± 0.1			
**Transferrin**				0.03	0.40	0.38
AA & GA	11.5 ± 2.4	12 ± 3	12.4 ± 3.2			
GG	12.1 ± 2.5	12.1 ± 3.2	12.6 ± 2.8			
**Transthyretin**				0.13	0.13	0.25
AA & GA	5.6 ± 1.2	5.6 ± 1.3	5.8 ± 1.4			
GG	5.6 ± 1.3	5.8 ± 1.3	5.9 ± 1.5			
**Vitamin D binding protein**				0.24	0.02	0.08
AA & GA	2.5 ± 0.4	2.6 ± 0.5	2.6 ± 0.5			
GG	2.7 ± 0.5	2.7 ± 0.6	2.7 ± 0.5			
**Vitronectin**				0.93	< 0.01	0.26
AA & GA	3.4 ± 0.6	3.5 ± 0.7	3.4 ± 0.7			
GG	3.6 ± 0.8	3.6 ± 0.8	3.6 ± 0.6			
**Zinc- α_2 -glycoprotein**				0.18	0.61	0.62
AA & GA	1 ± 0.4	1 ± 0.4	1.1 ± 0.4			
GG	1 ± 0.4	1 ± 0.4	1.2 ± 0.4			

Adjusted for Age, ethnicity, log BMI, sex, and physical activity.

Values are mean protein concentration ±standard error listed in order of increasing prudent dietary score; p-values are for linear regression models with protein concentrations as the dependent variable and prudent dietary pattern and binary genotype as the main determinants of interest as well as diet-gene as the interaction term.
